# KIT ligand produced by limbal niche cells under control of SOX10 maintains limbal epithelial stem cell survival by activating the KIT/AKT signalling pathway

**DOI:** 10.1111/jcmm.15830

**Published:** 2020-09-11

**Authors:** Zhongyuan Su, Jing Wang, Qinghua Lai, Huanyu Zhao, Ling Hou

**Affiliations:** ^1^ Laboratory of Developmental Cell Biology and Disease School of Ophthalmology and Optometry and Eye Hospital Wenzhou Medical University Wenzhou China; ^2^ State Key Laboratory of Ophthalmology, Optometry and Vision Science Wenzhou China

**Keywords:** KITL, limbal epithelial stem cells, limbal niche cells, neural crest, Sox10, stem cells, survival

## Abstract

Homeostasis and function of limbal epithelial stem cells (LESCs) rely on the limbal niche, which, if dysfunctional, leads to limbal epithelial stem cell deficiency (LSCD) and impaired vision. Hence, recovery of niche function is a principal therapeutic goal in LSCD, but the molecular mechanisms of limbal niche homeostasis are still largely unknown. Here, we report that the neural crest transcription factor SOX10, which is expressed in neural crest‐derived limbal niche cells (LNCs), is required for LNCs to promote survival of LESCs both in vivo and in vitro. In fact, using mice with a *Sox10* mutation and in vitro coculture experiments, we show that SOX10 in LNCs stimulates the production of KIT ligand (KITL), which in turn activates in LESCs the KIT‐AKT signalling pathway that protects the cells against activated CASPASE 3‐associated cell death. These results suggest that SOX10 and the KITL/KIT‐AKT pathway play key roles in limbal niche homeostasis and LESC survival. These findings provide molecular insights into limbal niche function and may point to rational approaches for therapeutic interventions in LSCD.

## 1 INTRODUCTION

Limbal epithelial stem cells (LESCs) play critical roles in regulating homeostasis of the corneal epithelium. Loss of LESCs leads to severe corneal disease, called limbal epithelial stem cell deficiency (LSCD), which is associated with corneal neovascularization, stromal scarring and impaired visual acuity.^1^ LESCs transplantation represents a feasible therapeutic approach to LSCD, but according to statistical analysis of clinical trials, this approach has nevertheless failed to improve vision in 26.7%‐60% of LSCD patients. These therapeutic failures may be due to the limited life span of LESCs.^2^, ^3^, ^4^, ^5^, ^6^, ^7^, ^8^, ^9^, ^10^, ^11^, ^12^ Evidently, a detailed analysis of the molecular mechanisms of LESC maintenance is of paramount importance to design approaches to enhance the success of therapeutic transplantation, or better still, to be able to interfere with LESC loss early in the disease process.^13^

The maintenance of adult stem cells relies on stem cell niches whose microenvironment contains an extracellular matrix and cells that provide autocrine and paracrine signals necessary for the proper function of the respective stem cells. There is increasing evidence that one of the major type of niche cells supporting LESCs are limbal niche cells (LNCs).^13^, ^14^, ^15^, ^16^, ^17^, ^18^, ^19^, ^20^, ^21^ Previous results have shown that LNCs support self‐renewal of LESCs,^21^ and LNCs transplants prevent LSCD in an alkali burn rabbit model.^22^ They likely do this by secreting paracrine factors including KITL,^22^ but the detailed mechanisms of KITL regulation in LNCs and how KIT, KITL and its downstream signal pathways are involved are not clearly understood. Here, we use both in vivo and in vitro approaches to provide insights into the regulation of KITL in LNCs and its action in LESCs.

Our studies were guided by the notion that LNCs are developmentally derived from neural crest cells (NCCs),^23^, ^24^ unlike LESCs, which are thought to be derived from the ocular ectoderm.^25^ In fact, besides LNCs, the neural crest contributes a number of cell types to the development and function of the eye. NCCs give rise, for instance, to the corneal endothelium, the corneal stroma and the stroma of the ciliary body and the iris. Not surprisingly, NCC deficiencies lead to corneal diseases, such as anterior segment dysgenesis (ASD),^26^, ^27^ as seen with Axenfeld‐Rieger syndrome,^28^, ^29^ Peters anomaly,^30^ Aniridia^31^ and Nail Patella syndrome.^32^ The major clinicopathological characteristics of ASD include corneal epithelial dystrophy, disorganized corneal stroma, sclerocornea, corneal opacities and corneal vascularization.^27^ Hence, NCCs are key cellular components of corneal development and homeostasis.

A major regulator of the NCC development is the transcription factor SOX10, an SRY‐box containing HMG DNA‐binding protein.^33^, ^34^ In humans, mutations in SOX10 are associated with several developmental NCC defects such as Waardenburg syndrome type IV (also known as Waardenburg‐Shah syndrome) and^35^ PCWH (peripheral demyelinating neuropathy, central dysmyelinating leukodystrophy, Waardenburg syndrome and Hirschsprung disease).^36^ Symptoms include, but are not limited to, deafness, skin pigmentary disorders and neurological defects. Interestingly, in vivo track systems based on SOX10‐cre‐driven YFP fluorescence have shown that SOX10‐positive NCCs are the major contributors to the corneal stroma.^24^

Given the above importance of SOX10 in NCCs and the fact that LNCs are essential for supporting self‐renewal of LESCs,^21^ we here focused on the role of SOX10 in LNCs for LESC maintenance both in vivo and in vitro. Using mice heterozygous for a *Sox10* mutation and isolated LNCs as well as their conditioned mediums, we show that a gene dose reduction of *Sox10* significantly impairs the ability of LNCs to support LESC maintenance and that SOX10 acts through KITL to activate the KIT‐AKT signalling cascade in LESCs. Hence, these findings suggest that the SOX10‐KITL/KIT axis is a major component of the supportive function of LNCs for LESCs.

## 2 MATERIALS AND METHODS

### 2.1 Animals

*Sox10^LacZ/+^* (hereafter called *Sox10/+*) mice, in which one *Sox10* allele is rendered non‐functional by insertion of a *LacZ* gene, were originally obtained from Dr Michael Wegner and then transferred to our laboratory from the laboratory of Dr William J. Pavan (NIH). Genotyping of *Sox10/+* mice was carried out as described.^37^ All animals were handled according to ethical standards of the Institutional Animal Care and Use Committee of the Wenzhou Medical University (permit number WZMCOPT‐090316).

### 2.2 Isolation and culture of both limbal niche cell and limbal epithelial stem cells

LESCs were isolated from 4‐week‐old mice by modifying a previously described method.^38^ Briefly, eyeballs of mice were washed with DMEM/F12 medium (Sigma‐Aldrich) containing 500 IU/mL penicillin (Beyotime Biotechnology) and 500 µg/mL streptomycin (Beyotime Biotechnology). Iris and excessive sclera were carefully removed, and limbal rings were isolated and incubated at 4°C for 16 hours with 1.2 IU/mL dispase II (Sigma‐Aldrich) dissolved in Hanks' balanced salt solution (Sigma‐Aldrich). Epithelial sheets were then carefully removed under a dissecting microscope, and single cell suspensions were prepared by treatment with 0.25% trypsin‐EDTA at 37°C for 5 minutes. Cells were collected by centrifugation at 400 *g* for 5 minutes and cultured in DMEM/F12 supplemented with 10% FBS (Invitrogen Corporation), 5 ng/mL recombinant mouse EGF (Sigma‐Aldrich), 1% ITS liquid media supplement (Sigma‐Aldrich), 0.5 µg/mL hydrocortisone (Solarbio), 30 ng/mL cholera toxin (Sigma‐Aldrich), 100 IU/mL penicillin and 100 µg/mL streptomycin.

LNCs were also isolated from 4‐week‐old mice as previously described,^14^ except for slight modification as follows: briefly, after limbal rings were isolated and epithelial sheets removed as mentioned above, the remaining limbal rings were cut into 1 mm^3^ pieces and incubated overnight at 4°C with DMEM/F12 medium containing 1 mg/mL collagenase A (Sigma‐Aldrich). After centrifugation, the pellets were resuspended in E8 medium (Life Technologies) and seeded onto 6‐well plates. Two days later, cell debris was carefully removed by aspirating the medium. Adherent LNCs usually grow out to form sphere‐shaped colonies 7 days after seeding. Both LNCs and LESCs were characterized by staining for differential expression of marker genes (Figure S1).

### 2.3 Colony formation assay

For preparation of conditioned medium, supernatants were collected from LNCs cultured with DMEM/F12 supplemented with 1% FBS for 3 days and then diluted at the ratio of 1:1 with DMEM/F12 medium containing 1% FBS. Similar procedures were used to prepare conditioned medium derived from LNCs transfected with si‐Sox10‐1, si‐Sox10‐2, si‐C (non‐specific siRNA used as a negative control) or mock‐transfected (hereafter called si‐Sox10‐1‐CM and si‐Sox10‐2‐CM, si‐C‐CMor mock‐CM, respectively). LESCs cultured with DMEM/F12 medium supplemented with 1% FBS served as controls.

For colony formation assays, 500 LESCs per well were seeded on the lower chambers of 24‐well cell culture inserts and cocultured with LNCs in the upper chambers. Alternatively, 500 LESCs were cultured on 24‐well cell culture plates and exposed to conditioned medium as mentioned above. Seven days after cell planting, a Giemsa Staining Kit (Sangon Biotech) was used to visualize colonies of LESCs according to the manufacturer's instructions. Efficiency of colony formation was determined by counting the numbers of colonies with radius greater than 0.2 mm and calculated as a percentage of the number of originally seeded cells.

To analyse the roles of KITL‐KIT signalling, 10 ng/mL KITL (R&D Systems), 1 µM imatinib mesylate as KIT inhibitor (Selleck Chemicals) or 0.2 µM AZD5363 as AKT inhibitor (Selleck Chemicals), were used during colony formation assays.

### 2.4 Structural analysis of cornea

For structural analyses of cornea, both WT and *Sox10/+* eyes were dissected, paraffin embedded, sectioned, and HE stained as described.^39^ Areas of the central cornea were photographed, and the thickness of the corneal epithelium was measured.

### 2.5 Real‐time polymerase chain reaction (RT‐PCR)

RT‐PCR was performed as previously described.^40^ Briefly, TRIzol reagent (Life Technologies) was used for total RNA extraction. Total RNA was then reverse‐transcribed into cDNA using a reverse transcriptase kit (Agilent), followed by RT‐PCR analysis using SYBR Green technology (Applied Biosystems). All data were normalized with respect to GAPDH expression levels. Sequences of primers used in this study are shown in Table S1.

### 2.6 siRNA transfection

To knock‐down gene expression in LNCs, siRNAs corresponding to sequences of the mouse *Sox10* gene were purchased from GenePharma. si‐C served as a negative control. Their sequences are as previously described.^40^ siRNAs were transfected into LNCs at a final concentration of 40 nM using LipoJet^TM^ In vitro Transfection Kit (SignaGen Laboratories) according to the manufacturer's instructions.

### 2.7 Immunostaining

For immunostaining, cells were fixed with 4% paraformaldehyde (PFA) for 20 minutes at room temperature and permeabilized with 0.4% Triton X‐100 for 10 minutes, followed by treatment of 1% BSA for 1 hour. Cells were then incubated with specific primary antibodies at 4°C overnight. Staining was revealed by either FITC or Cy3‐conjugated secondary antibodies. Photographs were taken using a Zeiss fluorescence microscope.

For immunocytochemical analysis, mice eyeballs were isolated and cryoprotected for preparation of frozen sections. Frozen sections were then immunostained as described above. The primary antibodies used in this study are shown in Table S2.

### 2.8 Western blot assay

Western blotting was carried out as described.^40^ Antibodies used in this study are shown in Table S3.

### 2.9 Enzyme‐linked immunosorbent assay (ELISA) and TUNEL assay

To detect the concentration of SCF in conditioned medium derived from LNCs, ELISA assays were carried out using SCF ELISA Kit (Invitrogen Corporation) according to the manufacturer's instructions.

To reveal apoptotic cells, terminal deoxynucleotidyl transferase dUTP nick end labelling (TUNEL) assay was carried out using In Situ Cell Death Detection Kit (Roche) according to the manufacturer's instructions.

### 2.10 Statistical analysis

Each experiment was repeated at least three times, and quantitative data are presented as mean ± SD. Statistical significance (*P*‐value) between experimental and control groups was assessed with Student's *t* test. *P* < .05 was considered statistically significant.

## 3 RESULTS

### 3.1 SOX10 controls the ability of LNCs to serve as a niche for LESC survival in vitro

**FIGURE 1:**
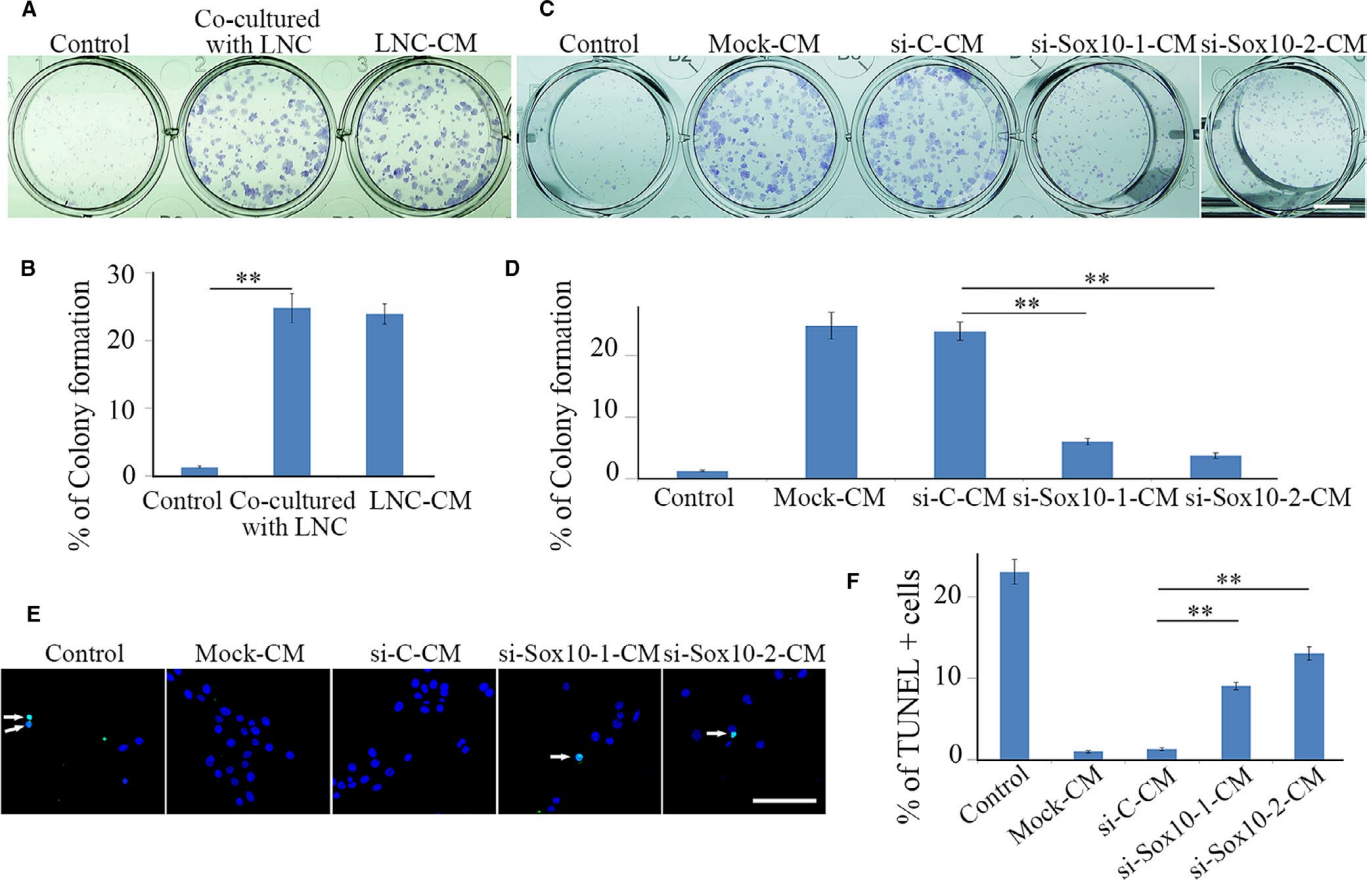
SOX10 is required for LNCs to support survival of LESCs in a paracrine fashion. (A), LNCs promoted colony formation of LESCs in a paracrine signal‐dependent manner. LESCs were cultured in 1% FBS (Control), cocultured with LNCs added to inserted chambers or cultured in LNC‐conditioned medium (LNC‐CM). LESCs colonies were visualized by Giemsa staining. Note that LNC‐CM promoted colony formation of LESCs, similar as LNCs did. (B), The percentage of LESC colonies was determined based on the results from A. (C), Knock‐down of Sox10 in LNCs diminished their supportive role for LESCs. Conditional medium was collected from LNCs after mock‐, si‐C, si‐Sox10‐1 or si‐Sox10‐2 transfection. The formation of LESCs colonies, defined as being >0.4 mm in diameter, was significantly reduced when cultured in si‐Sox10‐1‐CM or si‐Sox10‐2‐CM as compared to si‐C. (D), The percentage of colonies of LESCs was determined based on the results from C. (E), Apoptotic cells were visualized by TUNEL assay and TUNEL‐positive signals were indicated by white arrows. (F), The percentage of apoptotic cells was determined based on the results from E. All data are from triplicate experiments and are represented as mean ± SD. **indicates *P* < .01. Bar (A, C) = 0.5 cm, Bar (E) = 50 µm

To experimentally manipulate and examine the role of LNCs and their ability to support the maintenance of LESCs, both LNCs and LESCs were isolated and separately cultured in 24‐well plates with transwell inserts. The cells were characterized by immunostaining as shown in Figure S1. LNCs are P63‐, K14‐ and K15‐negative but VIMENTIN‐positive, while LESCs are P63‐, K14‐ and K15‐positive but VIMENTIN‐negative. Compared with control cell cultures in 1% FBS, colony formation was significantly increased when LESCs were cocultured with LNCs. Similar results were obtained when LESCs were cultured with conditioned medium derived from LNCs (LNC‐CM) (Figure 1A,B), lending support to the idea that LNCs act in a paracrine fashion to support LESCs.

As previously reported, LNCs originate from the neural crest and exhibit some characteristics resembling NCCs.^21^ Little, however, is known about the roles of neural crest signature genes in LNCs and their actions to sustain a functional limbus and cornea. Among these NC signature genes, *Sox10* encodes a well‐known transcriptional factor that is indispensable for NCC development and survival. Considering the fact that SOX10 is also highly expressed in LNCs (Figure S1), we hypothesized that SOX10 could orchestrate gene expression in LNCs to maintain a favourable microenvironment for LESCs. To test this hypothesis, siRNA was employed to knock‐down the expression of *Sox10* in LNCs. The results show that, compared with mock or control si‐C transfected LNCs, significantly lower expression levels of Sox10 mRNA and protein were detected in LNCs transfected with either si‐Sox10‐1or si‐Sox10‐2 (Figure S2). Compared with LESCs cultured in LNC‐CM, LESC colony formation was remarkably reduced when the cells were cultured with si‐Sox10‐1‐CM or si‐Sox10‐2‐CM (Figure 1C,D). The reduction in colony formation was accompanied by a significant increase in the number of apoptotic LESCs (Figure 1E,F) but only mildly decreased cell proliferation rates (Figure S3). These results suggest that SOX10 is needed for LNCs to maintain favourable niche conditions for LESC survival.

### 3.2 SOX10 in LNCs is required for survival of LESCs in vivo

**FIGURE 2:**
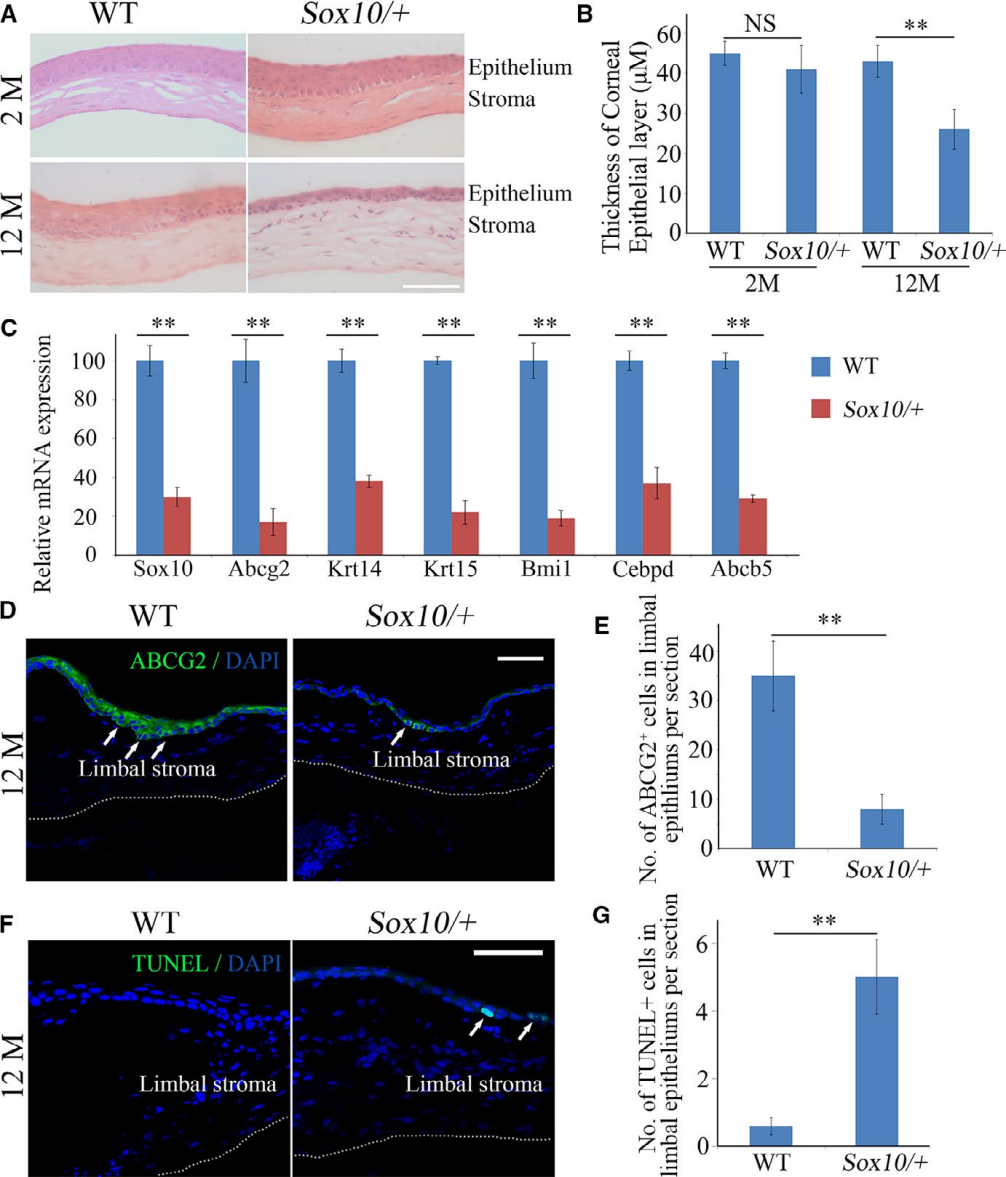
Sox10 is indispensable for the maintenance of both the corneal and limbal epithelium in vivo. (A), Down‐regulation of SOX10 impaired long‐term maintenance of the corneal epithelium. Note that, compared with WT littermates, the corneal thickness was reduced in 12‐month‐old *Sox10/+* mice. Bar = 100 µm. (B), The thickness of the corneal epitheliums was determined based on the results from A. (C), Expression of signature genes in limbal epithelial stem cells derived from either WT or *Sox10/+* limbus was assayed by RT‐PCR. Note that, compared with that of WT littermates, the expression levels of signature genes were reduced in *Sox10/+* limbus. (D), Limbal epithelial stem cells were visualized by immunostaining for ABCG2 in both WT and *Sox10/+* limbus. ABCG2^+^ cells are indicated by white arrows. (E), The number of ABCG2^+^ cells in the corneal epithelium was counted based on the results from D. Note that the numbers of ABCG2^+^ LESCs are remarkably decreased in *Sox10/+* mice. (F), Apoptotic cells in the limbal epithelium of both WT and *Sox10/+* were identified by TUNEL assays. Green fluorescence TUNEL‐positive signals are indicated by white arrows. (G), The number of apoptotic cells in the limbal epithelium were counted based on the results from F. Note that many more apoptotic cells were observed in the *Sox10/+* limbal epithelium. All data are from triplicate experiments and are represented as mean ± SD. **indicates *P* < .01. Bar (D, F) = 50 µm

Because embryonic or perinatal lethality in homozygous *Sox10*‐null mice precludes an analysis of long‐term corneal maintenance, we used *Sox10/+* mice to determine whether SOX10 controls LNCs to impact LESCs survival in vivo. Compared with their wild‐type (WT) littermates, 2‐month‐old *Sox10/+* mice did not show any obvious abnormalities in the cornea, but at 12 months, they displayed severe corneal dystrophy marked by reduced thickness of corneal epitheliums and disorganized corneal stroma (Figure 2A,B). These results indicate that heterozygosity for a Sox10 loss‐of‐function allele leads to progressive corneal epithelial dystrophy in mice. Since LESCs, which are self‐renewing, are instrumental in maintaining corneal physiology, we examined by RT‐PCR whether *Sox10/+* mice would show changes in marker genes in LESCs in the limbus. Compared with WT limbus, the expression levels of the LESCs marker genes *Abcg2, Krt14, Krt15, Bmi1, Abcb5* and *Cebpd* were reduced in the *Sox10/+* limbus (Figure 2C). A significant loss of ABCG2‐positive LESCs in the *Sox10/+* limbal epithelial layer was also detected by immunostaining (Figure 2D,E), accompanied by increased numbers of apoptotic cells in the limbal epithelium (Figure 2F,G), suggesting that LESCs are under apoptotic stress following a reduction in SOX10 gene dose in LNCs. Combined with the above‐mentioned in vitro data indicating that a reduction in SOX10 reduces LESC colony formation and survival (Figure 1C,D), it hence appears that down‐regulation of SOX10 contributes to a disruption of the niche for LESCs, which eventually leads to the reduction in the number of LESCs and to corneal epithelial dystrophy.

### 3.3 SOX10 regulates the expression of KITL in vitro and in vivo

**FIGURE 3:**
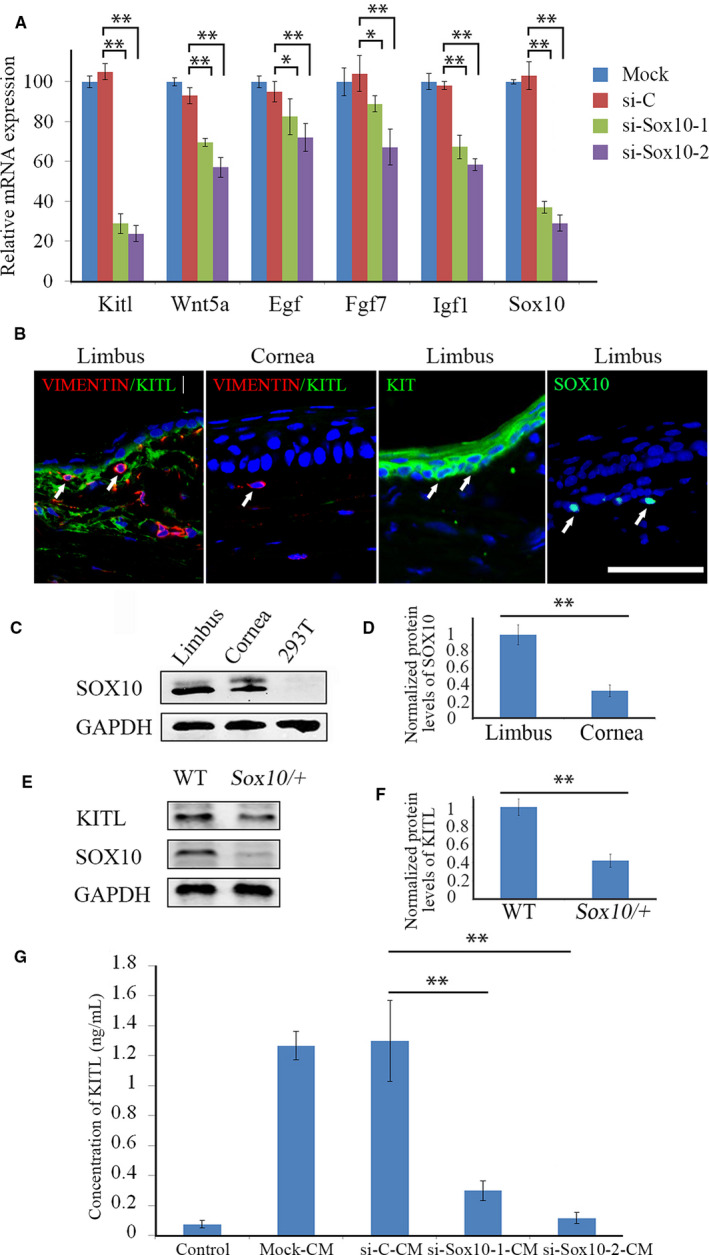
SOX10 regulates KITL expression in vitro and in vivo. (A), Knock‐down of Sox10 led to down‐regulation of the expression of a series of growth factor‐related genes in LNCs in vitro. LNCs were mock‐transfected or transfected with si‐C, si‐Sox10‐1 or si‐Sox10‐2. Note that the expression of *Kitl* was dramatically reduced when Sox10 was knocked down in LNCs. (B), Expression of KITL, KIT, VIMENTIN and SOX10 were visualized by immunostaining in LNCs in vivo and positive cells marked by white arrows. Note that, compared with LNCs, corneal stromal cells expressed significantly lower levels of KITL. Bar = 50 µm. (C), Expression of SOX10 in cornea or limbus was examined by Western blotting. HEK293T (293T) cells served as a negative control. (D), Normalized expression of SOX10 with respect to GAPDH was determined based on the results from C. Note that, compared with limbus, reduced expression levels of SOX10 were observed in cornea, similar to the reduced expression of KITL. (E), Expression of KITL in WT or *Sox10/+* limbus was examined by Western blotting. (F), Normalized expression of KITL with respect to GAPDH was determined based on the results from E. Compared with WT limbus, expression levels of KITL were down‐regulated in *Sox10/+* limbus. (G), Secreted isoform of KITL derived from LNCs was detected by ELISA in vitro. Note that, compared with either mock‐ or si‐C transfected LNCs, both si‐Sox10‐1‐ and si‐Sox10‐2‐transfected LNCs expressed remarkably lower levels of secreted isoform of KITL. All data are from triplicate experiments and are represented as mean ± SD. *indicates *P* <.1, **indicates *P* < .01

Growth factors are key components for the development and function of the limbal niche and accumulating evidence suggested that they play important roles in regulating LESCs survival and development.^22^, ^38^, ^41^, ^42^, ^43^, ^44^, ^45^, ^46^ In addition, as shown above, LNCs act in a paracrine fashion to support LESCs in vitro, and so, we decided to analyse whether SOX10 acts through growth factors to influence the survival of LESCs in LNCs. To identify which factors might be involved, we compared the expression of a series of growth factor genes in si‐Sox10 transfected LNCs vs si‐C or mock‐transfected LNCs. As shown in Figure 3A, knock‐down of *Sox10* led to down‐regulation of *Egf*, *Fgf7*, *Igf1*, *Kitl*, and *Wnt5a*. Among these genes, *Kitl* (also known as Stem cell factor, *Scf* or mast cell growth factor, *Mgf*) was of particular interest because it plays a pivotal role in LNCs to promote survival of LESC transplants.^22^ Hence, we examined the expression of both KITL and its receptor, KIT, by immunostaining. As shown in Figure 3B, KITL was highly expressed in the areas close to VIMENTIN‐positive LNCs in limbus, suggesting that KITL is involved in LESC maintenance. Expression of SOX10 was also detected in areas where LNCs are found (Figure 3B). Interestingly, compared with limbal cells, expression of KITL was hardly detected in corneal cells (Figure 3B), consistent with their reduced expression of SOX10 (Figure 3C,D). As shown in Figure 3E,F, Western blots indicated that the expression levels of both SOX10 and KITL were significantly reduced in limbus of *Sox10/+* as compared to WT mice. To further confirm that SOX10 has the ability to reshape the limbal niche by regulating the expression of the secreted isoform of KITL, we used ELISA assays to determine the protein levels of KITL in LNC‐conditioned medium. These assays indeed indicated increased levels of KITL in conditioned medium as compared to control medium or to medium derived from LNCs transfected with si‐Sox10 (Figure 3G). These results suggest, therefore, that SOX10 acts through KITL to maintain LESCs.

### 3.4 SOX10 works through activation of the KITL/KIT‐AKT signalling cascade to promote survival of LESCs

**FIGURE 4:**
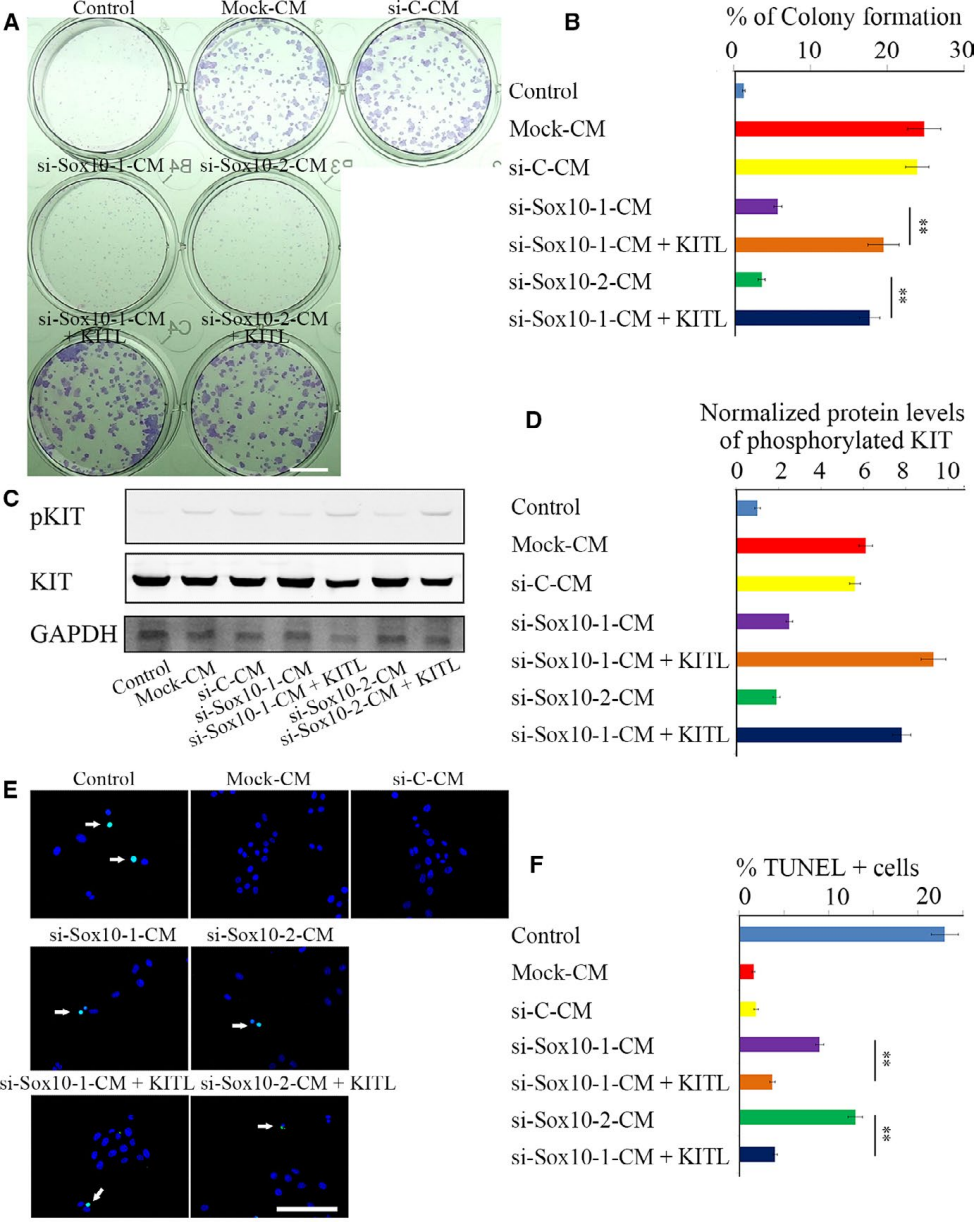
KITL partially rescues the survival of LESCs in Sox10‐knock‐down LNCs. (A), Colony formation assay. Colony formation of LESCs was recorded at day 7 post‐cell planting. Bar = 0.5 cm. (B), Colony formation of LESCs was determined based on the results from A. Note that, compared with that of LESCs cultured with si‐Sox10‐1‐CM or si‐Sox10‐2‐CM, the percentage of LESC colonies was significantly increased when a recombinant KITL was supplied. (C), Phosphorylation levels of KIT in LESCs were examined by Western blotting. (D), Normalized expression of phosphorylated KIT with respect to GAPDH was determined based on the results from C. Note that phosphorylation levels of KIT were up‐regulated in LESCs when KITL was supplemented. (E), KITL inhibits apoptosis of LESCs. Cell apoptosis was determined by TUNEL assay. Green fluorescence signal marked by white arrows represents TUNEL‐positive cells. Note that, compared with the numbers of apoptotic LESCs cultured with si‐Sox10‐1‐CM or si‐Sox10‐2‐CM, those obtained after supplementation of the CM with KITL were significantly reduced. Bar = 50 µm. (F), The ratios of apoptotic cells were determined based on the results from D. All data are from triplicate experiments and are represented as mean ± SD. **indicates *P* < .01

To determine whether SOX10 indeed acts through KITL produced by LNCs to support survival of LESCs, we first used recombinant KITL to treat cultures of LESCs. As mentioned, LESCs colony formation was diminished, and apoptosis enhanced, when the cells were exposed to si‐Sox10‐1‐CM or si‐Sox10‐2‐CM. Addition of recombinant KITL to such conditioned medium, however, significantly enhanced LESC colony formation and reduced cellular apoptosis (Figure 4A,D,E,F). Furthermore, Western blotting analysis revealed that the phosphorylation levels of KIT in LESCs were significantly increased provided the cells were exposed to a source of KITL either from LNC‐CM or from addition of recombinant KITL to si‐Sox10‐CM (Figure 4B,C). These results suggest that SOX10 works through KITL produced by LNCs to support LESCs survival.

**FIGURE 5:**
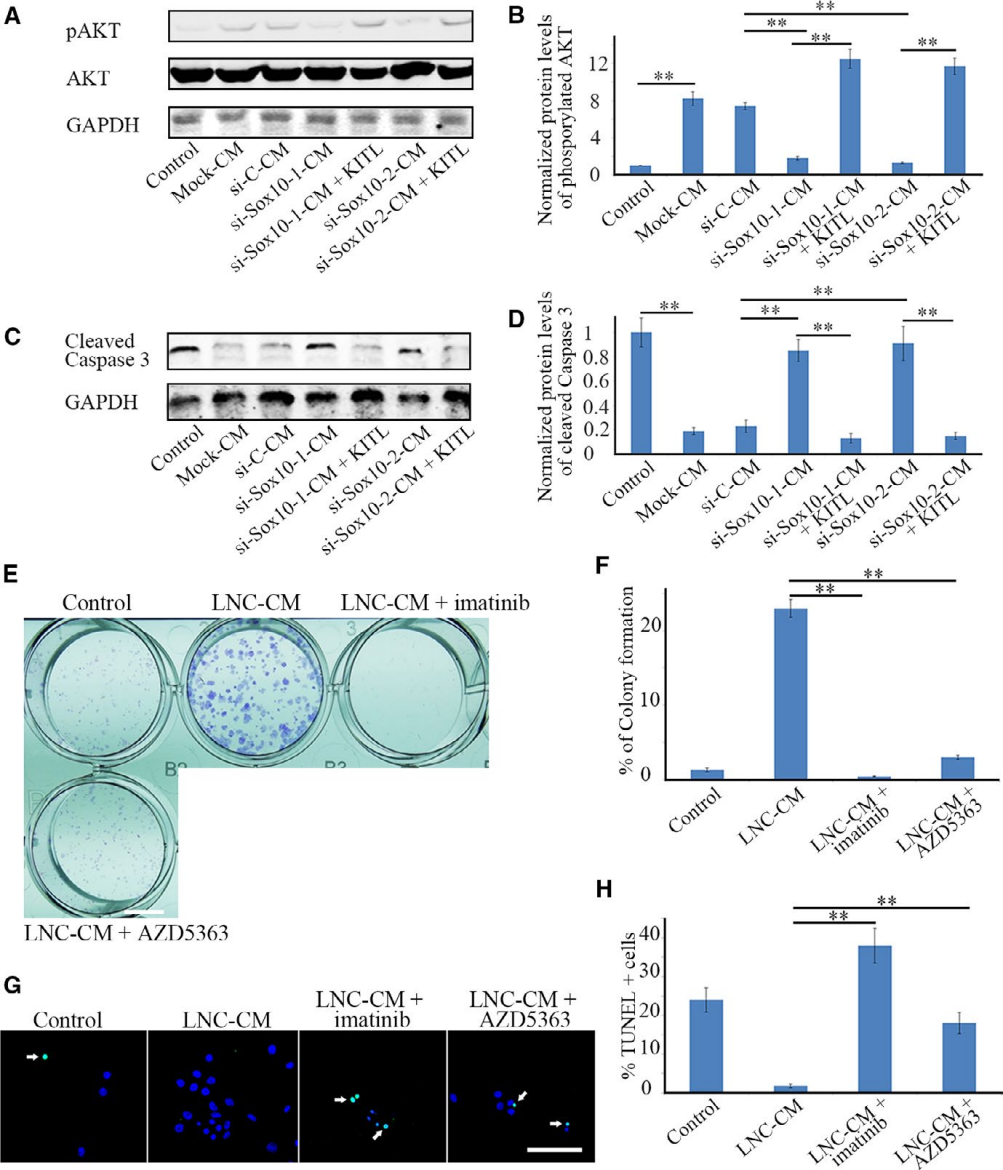
KITL/KIT signalling acts through activation of AKT to regulate the survival of LESCs. (A), Phosphorylation levels of AKT of LESCs were examined by Western blotting 7 d post‐cell planting. (B), Normalized expression of phosphorylated AKT was determined based on the results from A. Note that, compared with that of LESCs cultured in mock‐CM or si‐C‐CM, phosphorylation levels of AKT were significantly reduced in LESCs cultured with si‐Sox10‐1‐CM or si‐Sox10‐2‐CM. This reduction was reversed by KITL supplementation. (C), Activation of CASPASE 3 in LESCs was examined by Western blotting. (D), Normalized expression of cleaved CASPASE 3 with respect to GAPDH was determined based on the results from C. Note that, compared with that of LESCs cultured with control medium, expression levels of cleaved CASPASE 3 were significantly reduced in LESCs cultured with mock‐CM or si‐C‐CM. Cleaved CASPASE 3 levels were remarkably increased when LESCs were cultured with si‐Sox10‐1‐CM or si‐Sox10‐2‐CM, but addition of KITL restored the reduction. (E), Colony formation of LESCs, under treatment of KIT inhibitor (imatinib mesylate) or AKT inhibitor (AZD5363), was detected by Giemsa staining. Note that inhibition of KIT or AKT remarkably impeded colony formation of LESCs. Bar = 0.5 cm. (F), Colony formation of LESCs was determined based on the results from C. (G), Numbers of apoptotic cells were increased in LESCs treated with either imatinib mesylate or AZD5363. Green fluorescence TUNEL‐positive signals are indicated by white arrows. Note that inhibition of KIT or AKT signalling remarkably compromised survival of LESCs. Bar = 50 µm. (H) The ratios of apoptotic cells were determined based on the results from E. All data are from triplicate experiments and are represented as mean ± SD. **indicates *P* < .01

One of the pathways activated by KITL/KIT signalling is the AKT pathway.^47^ To address the question of whether SCF/KIT signalling acts through activation of AKT, Western blotting was carried out to detect the phosphorylation levels of AKT in LESCs. As shown in Figure 5A,B, compared with control cells, the AKT phosphorylation in LESCs was significantly increased when the cells were cultured with LNC‐CM, but not when they were cultured with si‐Sox10‐CM; addition of KITL to the latter conditioned medium, however, increased AKT phosphorylation again. Apparently, the activation pattern of AKT in LESCs parallels that of KIT (Figure 4B,C), indicating that SOX10‐stimulated expression of KITL in LNCs activates the KIT‐AKT signalling cascade in LESCs.

As AKT signalling has been shown to control the activation of CASPASE 3 to impact cell apoptosis,^48^ we examined whether AKT signalling would protect LESC from apoptosis by inhibiting CASPASE 3 cleavage. Using Western blots, we found, indeed, that the presence of KITL either in LNC‐CM or KITL‐reconstituted si‐Sox10‐CM significantly reduced cleaved CASPASE 3 levels (Figure 5C,D). These results suggest that SOX10 in LNCs acts through the KITL/KIT‐AKT signalling pathway to reduce activation of CASPASE 3 in LESCs. To further confirm the role of the KIT‐AKT signalling cascade in LESC survival, we treated LESCs with a KIT inhibitor, imatinib mesylate or an AKT inhibitor, AZD5363. Both inhibitors reduced the phosphorylation levels of AKT (Figure S4), increased LESC apoptosis and reduced LESC colony formation (Figure 5E‐H). Hence, Sox10‐mediated expression of KITL in LNCs and activation of the KIT/AKT signalling pathway in LESCs is essential for LESC survival.

## 4 DISCUSSION

The interest in LESCs is based on their importance for the development and physiology of the cornea whose structural integrity and translucency has to be maintained for life. In fact, loss or dysfunctions of LESCs can lead to disorganized corneal stroma and corneal epithelial dystrophy and are associated with a number of eye diseases. The replacement of these cells by transplantation, though feasible, has unfortunately not uniformly led to clinical improvements in affected patients.^3^ One of the reasons for the therapeutic failures may be the loss or instability of the transplanted cells. It is therefore imperative to delineate conditions under which the therapeutic success may be enhanced.

The development and maintenance of LESCs depends on interactions with neural crest‐derived LNCs, which, along with other cell types, form a stem cell niche providing trophic support for LESCs. Here, we used both genetic in vivo as well as in vitro approaches to examine molecular parameters of LNC‐mediated LESC survival. We find that the transcription factor SOX10, known to be instrumental in neural crest cell development, is expressed in LNCs, regulates their development and function^33^ and cell‐autonomously stimulates a number of growth factor genes, all of which potentially important for LESC survival. Among them we find KITL, known to be critical for the development of a number of neural crest derivatives, to be particularly well stimulated by SOX10. In fact, we find that upon interaction with its single receptor KIT present on LESCs, KITL leads to a paracrine activation of the AKT signalling pathway that in turn prevents apoptosis of LESCs. These results extend previous observations supporting a role for LNCs and KITL signalling in maintaining LESCs.^21^, ^22^

The above notion is based on multiple observations. First, both in vivo and in vitro, SOX10 is expressed in LNCs but not LESCs. Second, KITL expression is reduced in LNCs of mice heterozygous for a SOX10 null allele and in cultured LNCs derived from them. Third, LESCs express KIT, the sole receptor for KITL. Fourth, exposure of LESCs to LNCs or LNC‐conditioned medium leads to KIT phosphorylation and to activation of the AKT signalling pathway and prevents LESC apoptosis. Fifth, application of conditioned medium from LNCs, whose SOX10 expression has been reduced by application of corresponding siRNAs, results in reduced KIT signalling in LESCs and increased LESC apoptosis, and these effects can be reversed by re‐addition of recombinant KITL. Further support of these results comes from the application of inhibitors of KIT and AKT.

That SOX10 is particularly important for LNC development and function is supported by its wider role in the physiology of neural crest cells.^33^, ^49^ In fact, loss of SOX10 leads to severe defects in neural crest derivatives.^34^ Under appropriate conditions, SOX10 may even reprogramme cells to express neural crest markers^50^ and be associated with malignant transformation, for instance of neural crest‐derived melanocytes.^51^ Nevertheless, it has to be kept in mind that SOX10 is not the only factor critical for LNCs' support of LESCs as there are also other genes, such as *Pax3* and *Tfap2*, that are important in NCC development. TFAP2, for instance, has been shown to directly regulate the expression of *Sox10*,^52^ and PAX3 could synergistically cooperate with SOX10 to regulate fate determination of NCCs.^53^ Thus, it will be crucial to extend our current studies to analyse other genes for synergistic interactions with *Sox10* in LNCs and their role in providing trophic support for LESCs.

Much as SOX10 is important in development, so is KIT signalling, and this not only in NCCs^34^ but also in a variety of other cell types.^47^ Hence, the importance of KIT signalling in LESCs is not further surprising. KIT signalling is required for glycogen metabolism of the corneal epithelium,^54^ and loss of either KITL or KIT significantly impairs corneal wound healing.^55^ Interestingly, KIT is not only expressed in LESCs but also in photoreceptors, lacrimal canaliculus epithelial stem cells and eye wall cells.^56^, ^57^, ^58^ These cells are all essential for sustaining the physiological functions of the eye and are located in close proximity to SOX10‐positive NCCs or their derivatives.^24^ Hence, the SOX10‐KIT‐AKT axis may have a role in eye development and function beyond its role in maintaining LESCs that we demonstrated here.

Our results suggest a critical role of the SOX10‐KITL signalling pathway in LNC for maintaining LESC survival. In addition, other signalling pathways including the BMP and WNT pathway have also been suggested to be essential for LNCs' roles in supporting LESCs.^59^ Hence, a detailed knowledge of the molecular parameters of how the stem cell niche supports LESCs may evidently be of chief importance to design rational therapies for LSCD, one of the major causes of reduced visual acuity and blindness. In fact, reconstruction of the niche for LESCs may be an attractive strategy to treat LSCD. Both LNCs and growth factors derived from LNCs, including KITL and PEDF, have been applied towards niche reconstruction, and results from these studies are encouraging.^22^, ^60^ Nevertheless, it is currently not clear whether deficiencies in the SOX10‐KITL signalling pathway in the niche only affects LESCs, or whether such deficiencies also feedback on the niche cells themselves, in particular on LNCs. Given that mice heterozygous for a loss‐of‐function mutation in *Sox10* develop corneal structural abnormalities relatively late in life and that homozygosity for such *Sox10* mutations leads to embryonic or perinatal lethality, it may become necessary to generate conditional niche cell‐specific *Sox10* knockouts. Such models would also lend themselves to exploration of therapeutic approaches such as adeno‐associated virus‐based long‐term expression of SOX10 for niche reconstruction in LSCD.

In sum, our results provide evidence that SOX10 and KITL enable LNCs in a limbal stem cell niche to provide trophic support for LESCs and that KITL acts through the KIT‐AKT signalling cascade in LESCs. These findings contribute to our understanding of the molecular mechanisms underlying limbal niche function. We hope that they also provide hints for potential therapeutic avenues for patients afflicted with the devastating visual dysfunctions associated with limbal stem cell deficiency.

## CONFLICT OF INTEREST

The authors state no conflict of interest.

## AUTHOR CONTRIBUTION

**Zhongyuan Su:** Conceptualization (equal); Data curation (equal); Formal analysis (equal); Funding acquisition (equal); Investigation (equal); Project administration (equal); Writing‐original draft (equal); Writing‐review & editing (equal). **Jing Wang:** Data curation (equal); Formal analysis (equal); Investigation (equal); Resources (equal). **Qinghua Lai:** Data curation (equal); Investigation (equal). **Huanyu Zhao:** Data curation (equal); Investigation (equal). **Ling Hou:** Conceptualization (equal); Formal analysis (equal); Funding acquisition (equal); Methodology (equal); Project administration (equal); Resources (equal); Supervision (equal); Writing‐original draft (equal); Writing‐review & editing (equal).

## Supporting information

Supplementary Material
