# Supplementary material for: KIT ligand produced by limbal niche cells under control of SOX10 maintains limbal epithelial stem cell survival by activating the KIT/AKT signalling pathway
Source: J Cell Mol Med. 2020 Sep 11;24(20):12020–31. doi: 10.1111/jcmm.15830 (PMC7579694; doi:10.1111/jcmm.15830)
Supplement: Supplementary file 1 — Supplementary Material [file JCMM-24-12020-s001.doc]

**Supplemental information**

**Table S1 Sequence information of primers used in this study.**

| Gene name | NCBI Gene ID | Forward primer | Reverse primer |
| --- | --- | --- | --- |
| Sox10 | 20665 | ACACCTTGGGACACGGTTTTC | TAGGTCTTGTTCCTCGGCCAT |
| Abcg2 | 26357 | GAACTCCAGAGCCGTTAGGAC | CAGAATAGCATTAAGGCCAGGTT |
| Krt14 | 16664 | AGCGGCAAGAGTGAGATTTCT | CCTCCAGGTTATTCTCCAGGG |
| Krt15 | 16665 | AGCTATTGCAGAGAAAAACCGT | GGTCCGTCTCAGGTCTGTG |
| Bmi1 | 12151 | ATCCCCACTTAATGTGTGTCCT | CTTGCTGGTCTCCAAGTAACG |
| Abcb5 | 77706 | GCAAATTCTGAAAGAACCAACGG | TCAGCAAAGCGGAATATCTCAAT |
| Cebpd | 12609 | CGACTTCAGCGCCTACATTGA | CTAGCGACAGACCCCACAC |
| Kitl | 17311 | GAATCTCCGAAGAGGCCAGAA | GCTGCAACAGGGGGTAACAT |
| Wnt5a | 22418 | CAACTGGCAGGACTTTCTCAA | CATCTCCGATGCCGGAACT |
| Egf | 13645 | AGCATCTCTCGGATTGACCCA | CCTGTCCCGTTAAGGAAAACTCT |
| Fgf7 | 14178 | CTCTACAGGTCATGCTTCCACC | ACAGAACAGTCTTCTCACCCT |
| Igf1 | 16000 | CTGGACCAGAGACCCTTTGC | GGACGGGGACTTCTGAGTCTT |
| Gapdh | 14433 | AGGTCGGTGTGAACGGATTTG | TGTAGACCATGTAGTTGAGGTCA |

Note that all primer sets were designed to detect mouse genes.

**Table S2 Information of antibodies used for immunostaining in this study.**

| Antibodies | Source |
| --- | --- |
| rabbit anti-mouse ABCG2 antibodies | Cell signaling Technology, Danvers, MA, USA |
| rabbit anti-mouse KIT antibodies | Cell signaling Technology, Danvers, MA, USA |
| rabbit anti-mouse VIMENTIN antibodies | Cell signaling Technology, Danvers, MA, USA |
| rabbit anti-mouse KERATIN 14 antibodies | Sigma-Aldrich, St. Louis, MO, USA |
| rabbit anti-mouse KERATIN 15 antibodies | Sigma-Aldrich, St. Louis, MO, USA |
| goat anti-mouse Ki67 antibodies | Santa Cruz Biotechnology, Dallas, TX, USA |
| goat anti-mouse SOX10 antibodies | Santa Cruz Biotechnology, Dallas, TX, USA |
| rabbit anti ΔP63 antibodies | MXB Biotechnologies, Fuzhou, China |
| goat anti-mouse KITL antibodies | R&D Systems, Minneapolis, MN, USA |
| mouse anti-mouse SOX10 antibodies | R&D Systems, Minneapolis, MN, USA |

Note that goat anti-mouse SOX10 antibodies (Santa Cruz Biotechnology, Dallas, TX, USA) were used for immunostaining of frozen sections.

**Table S3 Information of antibodies used for Western blotting in this study.**

| Antibodies | Source |
| --- | --- |
| goat anti-SOX10 antibodies | Santa Cruz Biotechnology, Dallas, Texas, USA |
| goatanti-GAPDHantibodies | Kangcheng Bio-Tech, Shanghai, China |
| goat anti-mouse SCF antibodies | R&D Systems, Minneapolis, MN, USA |
| rabbit anti-mouse KIT antibodies | Cell signaling Technology, Danvers, MA, USA |
| rabbit anti-mouse phospho-KIT antibodies | Cell signaling Technology, Danvers, MA, USA |
| rabbit anti AKT antibodies | Cell signaling Technology, Danvers, MA, USA |
| rabbit anti phosphor-AKT antibodies | Cell signaling Technology, Danvers, MA, USA |
| rabbit anti-cleaved Caspase 3 antibodies | Cell signaling Technology, Danvers, MA, USA |

**SUPPLEMENTAL FIGURES and FIGURE LEGENDS**


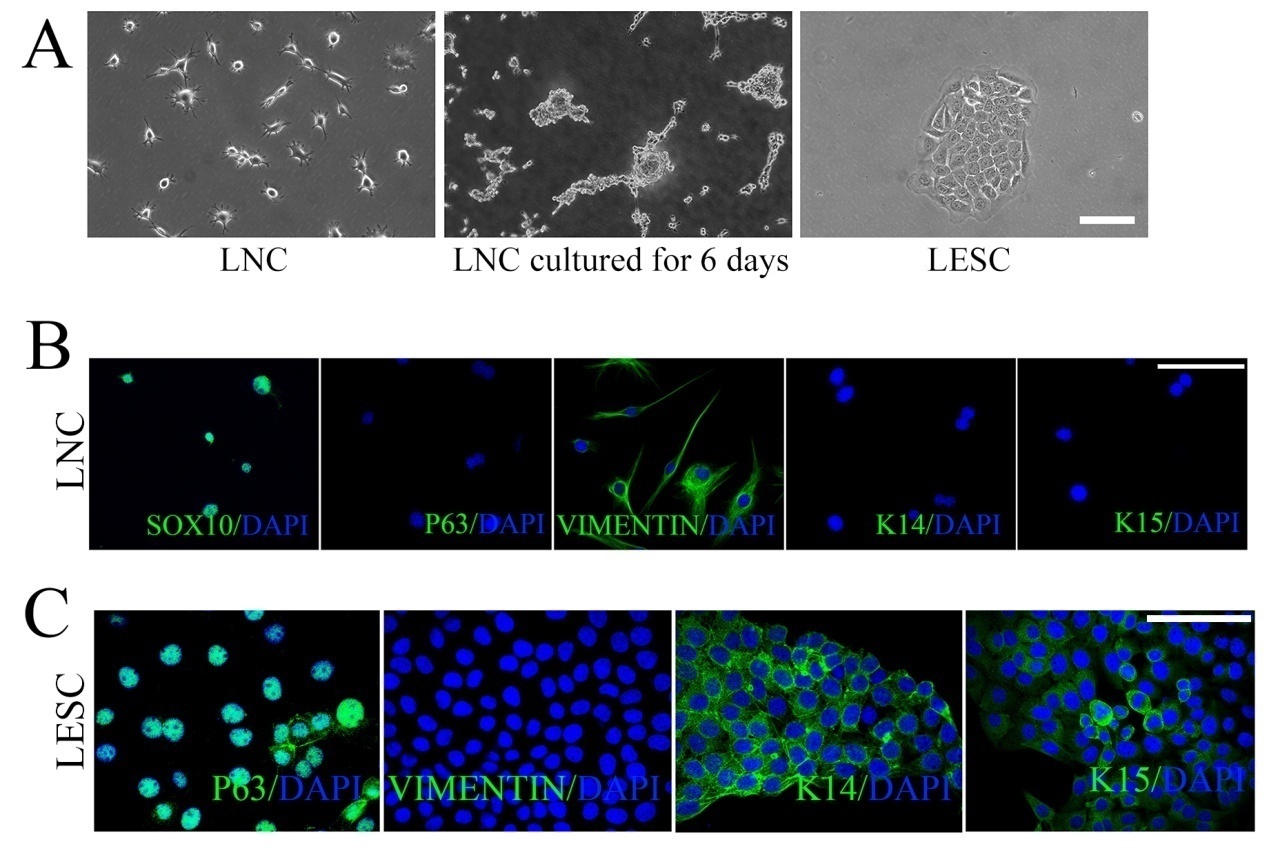


**FIGURE S1 Characterization of both LNCs and LESCs in culture.** (A), Representative images of LNCs or LESCs in culture. (B), Representative immunostaining images for LNCs markers. Note that LNCs were positive for SOX10 and VIMENTIN, but negative for the epithelial markers P63, KERATIN 14 (K14) and KERATIN 15 (K15). (C), Representative immunostaining images for LESC markers. Note that LESCs were positive for the epithelial markers P63, K14 and K15 but negative for VIMENTIN. Bar = 50 μm.


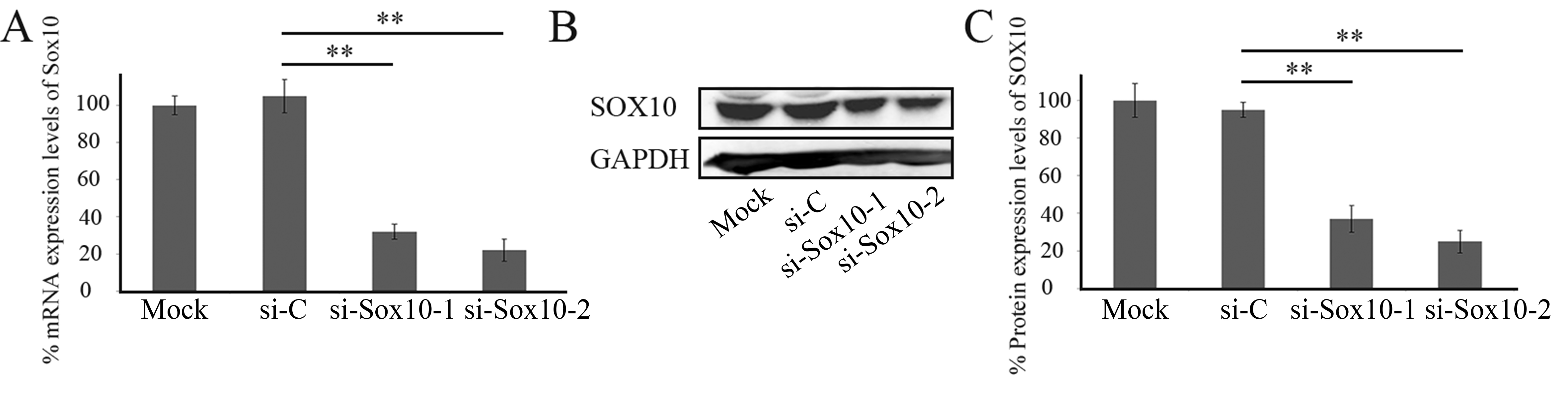


**FIGURE S2 Efficient reduction of Sox10 mRNA and protein expression by RNA interference in LNCs.** (A), LNCs cells were transfected with control siRNA (si-C) or siRNA specific for Sox10 (si-Sox10-1 and si-Sox10-2) and mRNA expression levels of Sox10 were measured by real-time PCR. (B), LNCs cells mock transfected or transfected with the indicated siRNAs were subjected to Western blot analysis. (C), Western blot results were quantified by density measurements of each band in (B) using the software of Bandscan. Note that Sox10 expression was significantly reduced by si-Sox10-1 or si-Sox10-2. ** indicates *P*<0.01.


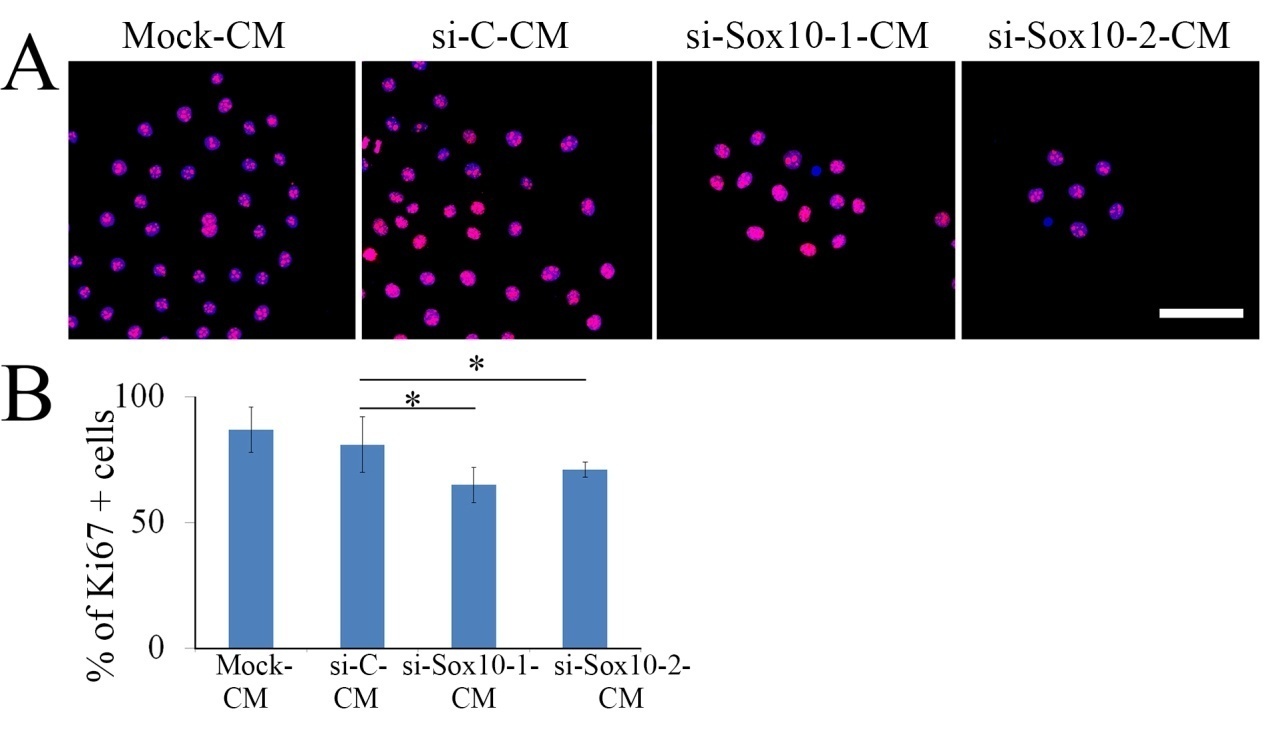


**FIGURE S3 Reduction of proliferation rate in cultured LESCs cultured with Sox10-knockdown medium.** (A), Representative immunostaining images for Ki67-positivity indicating proliferative cells. (B), The ratios of Ki67-positive cells were determined based on data from A. Data are from triplicate experiments and are represented as mean ± SD. * indicates *P* <0.1.Bar = 50 μm.


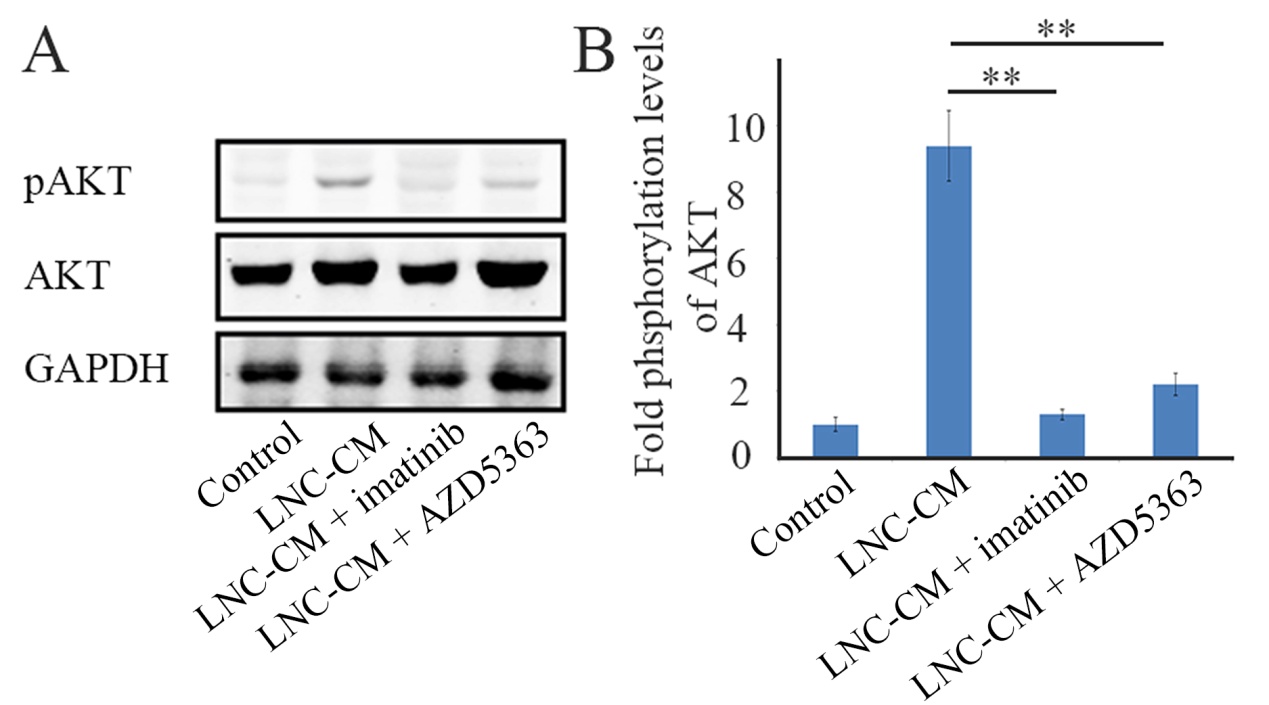


**FIGURE S4 Reduction of phosphorylation levels of AKT by either KIT inhibitor (imatinib mesylate) or AKT inhibitor (AZD5363).** (A), LESCs, cultured with LNC-CM, were treated with either 1μM imatinib mesylate or 0.2 μM AZD5363 for 30 minutes, and phosphorylation levels of AKT were analyzed by western blotting. (B), Expression of phosphorylated AKT, normalized with respect to GAPDH expression, was determined based on data from A. Data are from triplicate experiments and are represented as mean ± SD. * *indicates *P*<0.05.
